# Recombinant human alpha fetoprotein synergistically potentiates the anti-cancer effects of 1′-S-1′-acetoxychavicol acetate when used as a complex against human tumours harbouring AFP-receptors

**DOI:** 10.18632/oncotarget.3951

**Published:** 2015-04-29

**Authors:** Norhafiza M. Arshad, Lionel L.A. In, Tchen Lin Soh, Mohamad Nurul Azmi, Halijah Ibrahim, Khalijah Awang, Elena Dudich, Eduard Tatulov, Noor Hasima Nagoor

**Affiliations:** ^1^ Institute of Biological Science (Genetics and Molecular Biology), Faculty of Science, University of Malaya, Kuala Lumpur, Malaysia; ^2^ Department of Biotechnology, Faculty of Applied Sciences, UCSI University, Kuala Lumpur, Malaysia; ^3^ Institute of Biological Science (Ecology and Biodiversity), Faculty of Science, University of Malaya, Kuala Lumpur, Malaysia; ^4^ Centre for Natural Product Research and Drug Discovery (CENAR) and Department of Chemistry, Faculty of Science, University of Malaya, Kuala Lumpur, Malaysia; ^5^ Centre for Research in Biotechnology for Agriculture (CEBAR), University of Malaya, Kuala Lumpur, Malaysia; ^6^ Institute of Immunological Engineering, Lyubuchany, Moscow, Russia

**Keywords:** acetoxychavicol acetate, alpha fetoprotein, anti-cancer, alpinia conchigera, targeted cytotoxicity

## Abstract

**Purpose:**

Previous *in vitro* and *in vivo* studies have reported that 1′-S-1′-acetoxychavicol acetate (ACA) isolated from rhizomes of the Malaysian ethno-medicinal plant *Alpinia conchigera* Griff (Zingiberaceae) induces apoptosis-mediated cell death in tumour cells via dysregulation of the NF-κB pathway. However there were some clinical development drawbacks such as poor *in vivo* solubility, depreciation of biological activity upon exposure to an aqueous environment and non-specific targeting of tumour cells. In the present study, all the problems above were addressed using the novel drug complex formulation involving recombinant human alpha fetoprotein (rhAFP) and ACA.

**Experimental Design:**

To study the synergistic effect of both agents on human cancer xenografts, athymic nude (*Nu/Nu*) mice were used and treated with various combination regimes intraperitoneally. Serum levels of tumour markers for carcinoembryonic antigen (CEA) and prostate specific antigen (PSA) were assessed using sandwich ELISA. IHC and Western blotting were also conducted on *in vivo* tumour biopsies to investigate the involvement of NF-κB regulated genes and inflammatory biomarkers. Quantification and correlation between drug efficacies and AFP-receptors were done using IF-IC and Pearson's correlation analysis.

**Results:**

Mice exposed to combined treatments displayed higher reductions in tumour volume compared to stand alone agents, consistent with *in vitro* cytotoxicity assays. Milder signs of systemic toxicity, such as loss in body weight and inflammation of vital organs were also demonstrated compared to stand alone treatments. Tumour marker levels were consistent within all rhAFP/ACA treatment groups where levels of CEA and PSA were initially elevated upon commencement of treatment, and consecutively reduced corresponding to a decrease in tumour bulk volume. Both IHC and Western blotting results indicated that the combined action of rhAFP/ACA was not only able to down-regulate NF-κB activation, but also reduce the expression of NF-κB regulated genes and inflammatory biomarkers. The efficacy of rhAFP/ACA complex was also found to be weakly negatively correlated to the level of surface AFP-receptors between tumour types.

**Conclusions:**

This drug complex formulation shows great therapeutic potential against AFP-receptor positive tumours, and serves as a basis to overcome insoluble and non-specific anti-neoplastic molecules.

## INTRODUCTION

Cancer is amongst one of the most challenging health problems in the world today. Even with advances in medical science disciplines such as surgery, immunotherapy, chemotherapy, hormonal therapy, and radiotherapy, there is still no significant progress in its treatment. The conventional radiotherapy and chemotherapy with synthetic drugs used in treating cancer are not only expensive, but also induce severe side effects including immunosuppression, organ failure and susceptibility toward infectious diseases which may cause the death of patients upon treatment [1]. Thus, more efficient and low cost drugs that evoke lower systemic toxicity via specific targeting of cancer cells would be desirable in cancer management and treatment. Strategies involving combined therapies or agents with distinct molecular mechanisms are considered more promising for higher efficacy and better survival. The rationale for combination therapy is to use drugs that work by different mechanisms of action to decrease the likelihood of resistant cancer cell development. As a consequence, there is an increase in the number of preclinical and clinical studies involving novel combinations of anti-cancer drug therapies and chemopotentiating agents to improve cancer treatment outcome [2].

We have previously demonstrated that 1′-S-1′-acetoxychavicol acetate (ACA) isolated from rhizomes of the Malaysian ethno-medicinal plant, *Alpinia conchigera* Griff (Zingiberaceae), induces apoptosis-mediated cell death on various cancer cell lines while minimally affecting HMEC normal cells *in vitro* and *in vivo* [3-4]. Additionally, ACA further enhanced the cytotoxic effects of cisplatin (CDDP) in a synergistic manner, acting as a chemopotentiator. Its combined effects with CDDP *in vivo* produce an improved chemotherapeutic regime with increased efficacies at lower concentrations, which could hypothetically reduce the occurrence of dose-limiting toxicities. We also found that the effects of ACA correlated with a down-regulation of NF-κB regulated genes (*FASL* and *BIM*), including proinflammatory (*COX-2*) and proliferative (*cyclin D1*) biomarkers in tumour tissues. Consequent to this, ACA was deemed to inhibit the constitutive activation of NF-κB through suppression of IKKα/β activation modulated through dysregulation of the NF-κB pathway [4].

Despite the strong molecular basis that showed ACA's capability to increase the cytotoxic efficacy on human tumour cell lines, there were some clinical development limitations such as poor solubility in aqueous solution, declining biological activity upon exposure to an aqueous environment and poor specific targeting of tumour cells for its therapeutic use *in vivo* making it less desirable for cancer treatment. These problems are common in polyphenols, isoflavons or flavonoids of plant-derived anti-tumour compounds which have extremely low solubility in water and require polar solvents, such as dimethyl sulfoxide (DMSO), ethanol or certain medicinal oils, to be soluble. Therefore, we sought to search for counter measures to address ACA's drawback as a potential chemotherapeutic agent.

Alpha fetoprotein (AFP) has been well known to bind and transport a multitude of ligands such as bilirubin, fatty acids, retinoids, steroids, hormones, flavonoids, phytoestrogens, heavy metals, dioxins and various organic drugs [5]. Functions of AFP includes, its use as growth and differentiation factors for embryonic stem cells and tissues [6], operates as a suppressive factor for tumours [7] or activated immune cells [8], and does not affect the proliferation of normal untransformed cells [9]. Since a large number of tumours express AFP-receptors in addition to normal hepatocytes, AFP could be seen as a death factor, highly selective against tumour cells but completely non-toxic to normal cells. It has been shown earlier that human AFP can be actively expressed in *E. coli* [10] as well as in yeast cells [11-12], with recombinant AFPs having biological properties related, but not identical to native human AFPs [11-12]. It was also shown that these recombinant human AFPs (rhAFP) acts as a non-covalent carrier for various active apoptosis-inducing water-insoluble ingredients aimed at cells expressing AFP receptors (AFPRs) [10, 13-14]. Thus, the concept of developing drug conjugates or complexes to optimize patients' tolerance and increasing the anti-tumour efficiency of common chemotherapeutic drugs via directed delivery of cytotoxic agents towards the target cells using protein molecules seems to be a very promising strategy.

Interactions of pharmaceutical drugs with serum constituents are an important issue in drug delivery. Transport protein molecules can be classified as specific selective carriers and unselective carriers, where the former should provide targeted delivery of cytotoxic ligands selectively to tumour cells while avoiding normal cells. Therefore, the objective of this study was to develop a complex formulation of ACA with a specific carrier such as rhAFP for selective targeting of tumour cells for clinical use, especially in injectable forms as the substance is practically insoluble in water. Development of non-covalent hydrophobic pocket interactions between the insoluble active ACA and rhAFP while optimizing their apoptotic and synergistic efficacies were evaluated in both *in vitro* and *in vivo* systems in comparison to stand alone treatments. The aim was to develop a protein carrier capable of targeting the delivery of the anti-cancer drugs selectively to tumour cells while avoiding normal healthy tissue, hence allowing minimization of non-specific toxicity.

## RESULTS

### Formation of rhAFP/ACA non-covalent complex

**Figure 1:**
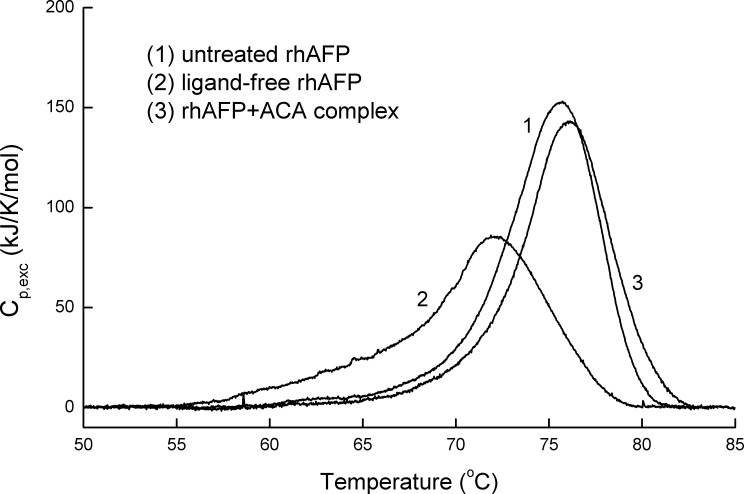
Calorimetric scan of the intact ligand-free rhAFP, rhAFP/ACA complex and rhAFP after ligand removal ACA removal drastically changes the melting pattern of rhAFP, while the addition of the ACA to rhAFP completely recovered the initial protein melting pattern which was characteristic of the intact rhAFP molecule. The protein concentration was 2.0 mg/ml in PBS, pH 7.4. Relative amount of ligands in multimolecular complex rhAFP/ACA was 1:1.

Previous research have shown that AFP can bind metals and small hydrophobic molecules while inducing significant stabilization of the tertiary structure of the protein with respect to thermal melting [12]. These conformational changes can be easily monitored by measuring the heat melting parameters, such as enthalpy of denaturation transition and temperatures, which are characteristic parameters for the conformational state of protein macromolecules. In this study, adiabatic scanning microcalorimetry technique was used to assess the interaction of hydrophobic ACA molecules, by forming a stable non-covalent complex with rhAFP as a carrier protein. Lipophilic drugs can enter the hydrophobic binding sites of rhAFP and affect thermodynamic parameters including the specific excess heat capacity, specific enthalpy of denaturation transition and temperature, thereby allowing the monitoring of ligand binding processes through the observation of conformational change manifestations. Information on the steric structure of formed complexes allows visualization on the conformational change in rhAFP molecules due to hydrophobic ACA binding, indicating the formation of a non-covalent complex (Figure 1). Thermodynamic parameters obtained for various rhAFP sample conditions showed that ligand removal led to a significant decrease in values of denaturation enthalpies, and transition temperatures are shown in Table 1. This indicates that changes in thermodynamic parameters of the rhAFP molecule induced by ACA removal reflected destabilization of the rhAFP tertiary structure. On the other hand, loading of rhAFP with ACA led to a notable increase in denaturation temperatures and enthalpy values of distinct transitions, showing stabilization of rhAFP's molecular structure in respect to heat melting.

**Table 1:** The thermodynamic effects of ligand-protein interaction on rhAFP heat melting parameters

Sample^†^	ΔH [kJ·mol-1]^††^	Tmax [ºC]^††^	Cooperativity [ºC]^‡^
Untreated rhAFP	970.2	75.7	7.5
Ligand-free rhAFP	757.6	72.1	7.1
rhAFP+ACA; rhAFP/ACA (1:1)	908.0	76.0	5.4

† Protein concentration was 2.0 mg/ml in 1×PBS, pH 7.4

†† Error in enthalpies are approximately ± 6%. Errors in denaturation temperatures are approximately ±0.5ºC

‡ Half width of the denaturation transition peak.

### *In vitro* cytotoxic effects of rhAFP/ACA complex

**Figure 2:**
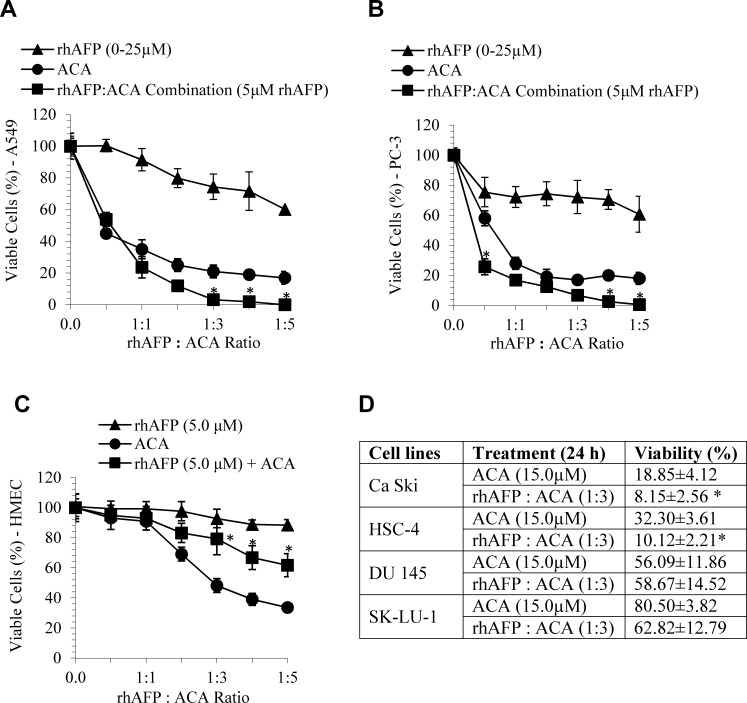
*In vitro* combined cytotoxic effects of ACA stand alone, rhAFP stand alone, and rhAFP/ACA complex The *in vitro* effects at various molar ratios after 24 h treatment against **A.** A549 human lung, **B.** PC-3 human prostate cancer cells, **C.** HMEC human mammary epithelial non-cancerous cell control, and **D.** summary of other human cancer cell lines tested. Data shown as mean ± S.D. of three independent replicates. Statistically significant differences between rhAFP+ACA values versus ACA stand alone values are marked by (*).

In order to evaluate the efficacy of ACA in combination with rhAFP, MTT assays were performed on six human cancer cell lines, which were NSCLC (A549 and SK-LU-1), prostate (PC-3 and DU 145), cervical (Ca Ski) and oral (HSC-4) cancer cells. The rhAFP/ACA complex was successful in increasing the cytotoxic efficacy in comparison to stand alone ACA treatments in most of the cell lines tested (Figure 2). Significant cytotoxic improvements were obtained in rhAFP/ACA treated PC-3 and A549 cells, with reductions of ~50.0% and ~25% respectively at ≥1:3 molar ratios in comparison to ACA stand alone treatments. The overall efficacy of combined rhAFP/ACA regime was found to be consistently improved between all other cell lines tested (with the exception of DU 145 and SK-LU-1 cells) within an effective therapeutic IC_50_ dose of 1.50 μM to 2.50 μM when used with a fixed rhAFP concentration of 5.0 μM (data not shown). This indicates that a low rhAFP dose notably enhances efficacy of ACA cytotoxicity allowing significant decrease of effective therapeutic doses, and possibly enhanced specificity towards cancer cells. When MTT assays employing various combination ratios of ACA with rhAFP ranging from 1:1 to 1:5 was carried out, we found that efficacy improvements in A549 cancer cells were more prominent at lower viability levels (<30%) and were more dependent on different molarity ratios compared to its effects on PC-3 cancer cells. When tested on HMEC normal mammary epithelial cells, where AFPRs are expected to be minimal or absent, the toxicity of ACA stand alone was clearly reduced in the presence of rhAFP, with a 30% increase in viability, suggesting the successful tumour targeting nature of rhAFP/ACA complex (Figure 2). *In vitro* cytotoxicity data also suggests that the molar ratio between rhAFP and ACA from 1:1 to 1:3 was a suitable combination range, and that further improvements on tumour suppression efficacies cannot be determined at ratios above 1:3 as there are no viable cells present (Figure 2).

### Potentiation of *in vivo* anti-tumour effects by rhAFP/ACA complex

**Figure 3:**
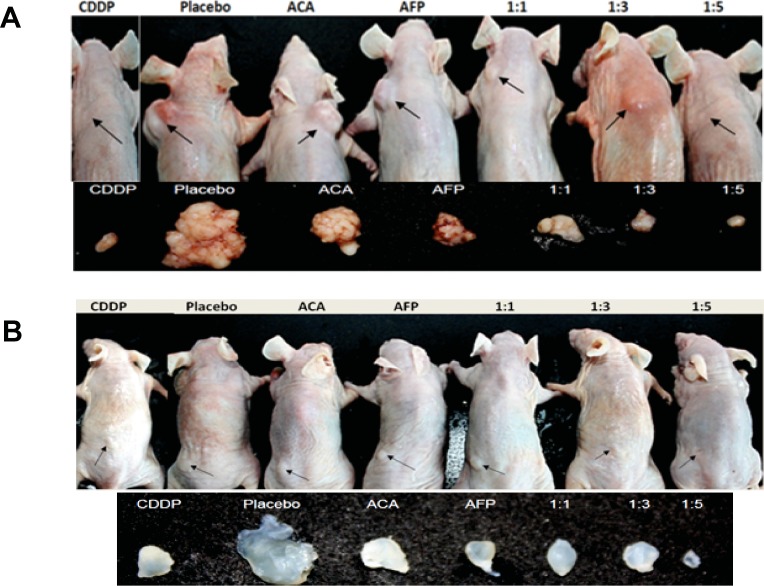
Tumour reduction effects of various rhAFP/ACA treatment regimes on *Nu/Nu* mice Location of all surface tumour sites are indicated by closed arrows, and representative photographs (*n*=6) of tumours harvested 35 days post-implantation for **A.** NSCLC A549 tumour xenografts and **B.** Prostate PC-3 tumour xenografts. Saline solution 0.9% (w/v) sodium chloride was used as placebo, while CDDP (10.0 mg/kg) was used as a positive control reference. Average tumour volumes for each treatment group are shown under Supplementary Figure 1.

Two groups of murine NSCLC and prostate cancer xenograft models were used to assess the anti-tumour therapeutic of rhAFP/ACA complex *in vivo*. We found that all molar ratios of rhAFP/ACA tested resulted in superior tumour volume regression compared to placebo, ACA stand alone, rhAFP stand alone and CDDP positive control groups (Figure 3). In the therapeutic group, molar ratios of 1:1 and 1:3 were found to be the most effective in reducing tumour volume, thereby also indicating that these regimens were the optimum complexing ratio between free hydrophobic pockets on rhAFP with insoluble ACA. It was also noted that when treatment was ceased, tumour volume manifested significantly comparable to placebo bulk volumes within just 2 weeks, presumably due to pre-mature regime termination resulting in an incomplete eradication of tumour remnants (Supplementary Figure 1).

### Physiological effects rhAFP/ACA treatment *in vivo*

**Figure 4:**
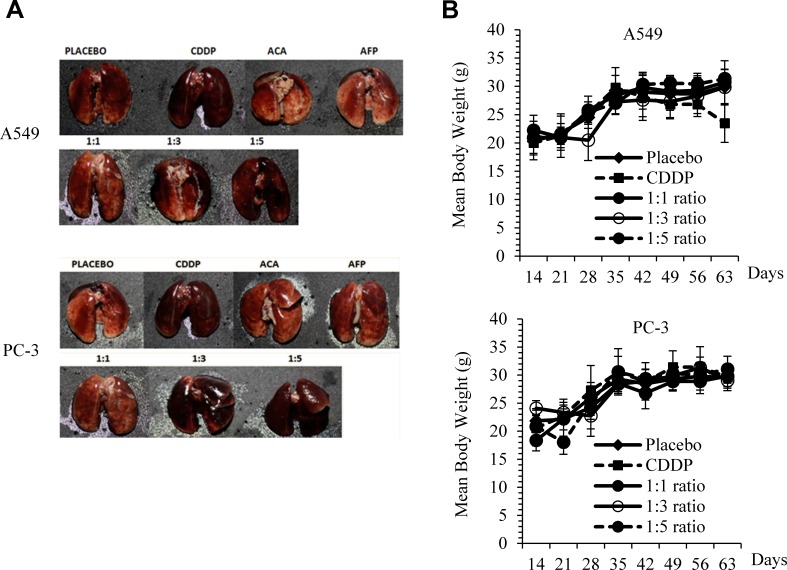
Physiological side effects of rhAFP/ACA *in vivo* **A.** Signs of pulmonary inflammation and capillary haemorrhaging in CDDP treated groups and at high rhAFP/ACA molar ratio regimes (≥1:3) compared to placebo in A549 human NSCLC and PC-3 human prostate cancer xenografts. **B.** Assessment on mean ± S.D. body weight loss between various combined rhAFP/ACA treatment groups on A549 and PC-3 xenografts. Placebo denotes groups treated with 0.9% (w/v) sodium chloride solution while concentration of CDDP was set at 10.0 mg/kg once per week over 4 weeks. Treatment commenced 2 weeks post-tumour implantation.

In assessing side effects, we found that signs of pulmonary inflammation were absent when a 1:1 molar ratio of rhAFP/ACA regime was used in comparison to CDDP treated mice. When a high molar ratio regimen (≥1:3) was employed, the presence of pulmonary inflammation and capillary haemorrhaging was noticeable in both A549 and PC-3 groups (Figure 4A). The activation of pro-inflammatory signalling in response to rhAFP/ACA was also found to be consistent with the activation of the NF-κB pathway, the major transcription factor family governing inflammatory cytokine responses, as shown later in Western blotting and IHC analyses. Body weight loss was apparent only in CDDP-treated mice, while all rhAFP/ACA treated groups maintained a similar increase in body weight comparable to placebo groups (Figure 4B). No other physiological indications on other major organs, such as liver and kidney were found during necropsy procedures. It was concluded that the inflammatory and physiological effects of rhAFP/ACA was somewhat milder than conventional CDDP drugs, which was likely attributed to rhAFPs tumour targeting abilities.

### Treatment with rhAFP/ACA reduces tumour marker levels *in vivo*

**Figure 5:**
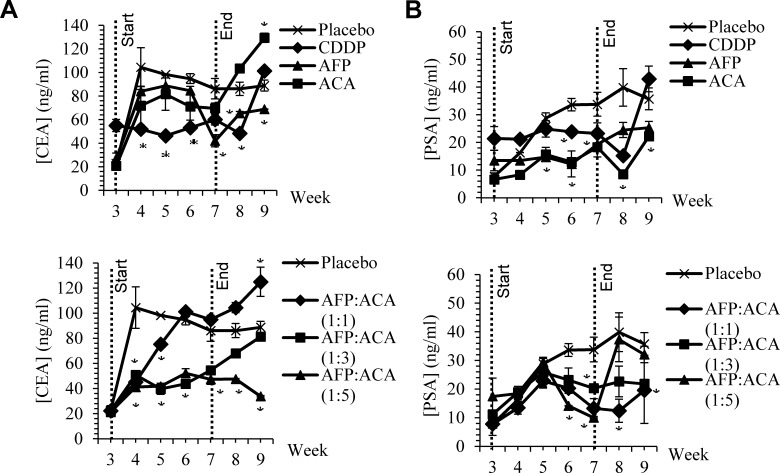
CEA and PSA tumour antigen marker levels Weekly CEA and PSA tumour antigen marker levels from blood sera of nude mice harbouring **A.** A549 human NSCLC tumour xenografts and **B.** PC-3 human prostate tumour xenografts upon treatment with rhAFP/ACA complex at various molar concentration ratios in comparison to placebo, stand alone and CDDP controls. Start and end of treatment is shown by dotted lines at week 3 and 7. All values shown are mean values ± S.D. Statistically significant changes against placebo groups are denoted as (*) with a *p* ≤ 0.05 threshold.

Monitoring of treatment effectiveness was also measured weekly using sandwich ELISA against tumour antigen markers. Carcinoembryonic antigen (CEA) was used to monitor the development of NSCLC tumours while prostate specific antigen (PSA) was used for assessing prostate tumours. In comparison to placebo controls, both PSA and CEA levels reduced consistently as rhAFP/ACA molar concentrations increased from 1:1 to 1:5 molar ratios (Figure 5). Reductions in tumour marker levels were also found to be short lived, and increased rapidly back when treatment ceased, consistent with changes in tumour bulk volume for all treated groups.

### rhAFP/ACA complex mediates its anti-cancer and anti-inflammatory effects through the NF-κB signalling pathway

**Figure 6:**
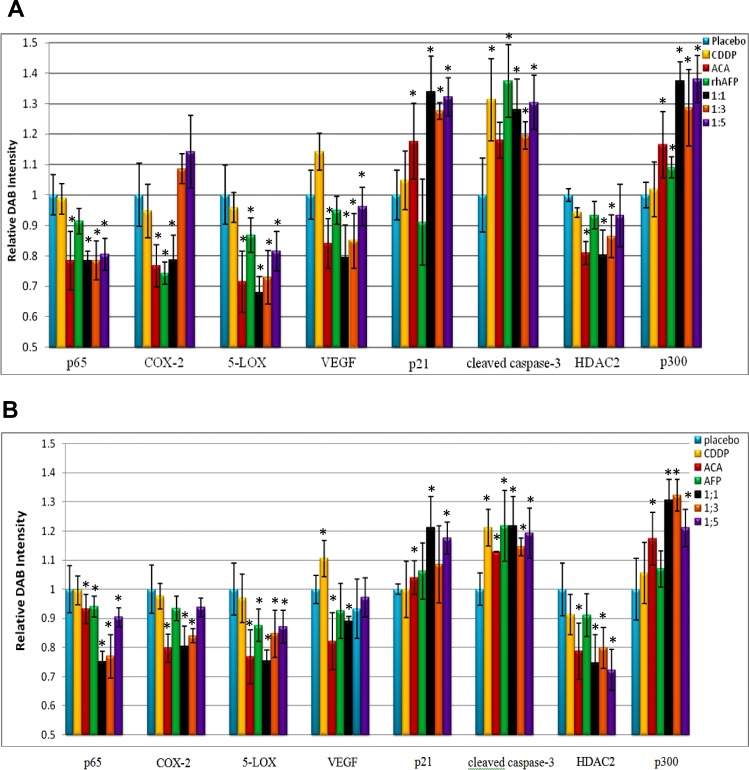
Quantification of IHC DAB relative intensity Quantification of IHC DAB relative intensity of **A.** A549 NSCLC xenograft sections and **B.** PC-3 prostate xenograft sections treated with various rhAFP/ACA combination regimes. Data for all NF-κB regulated proteins and inflammatory biomarkers were presented as mean ± S.D. of three independent replicates. Statistically significant changes against placebo groups are denoted as (*) with a *p* ≤ 0.05 threshold.

In previous *in vitro* analyses, it was reported that both ACA and rhAFP mediates their anti-cancer effects through the NF-κB signalling pathway and by disrupting XIAP-caspase interaction respectively [4, 17]. In order to determine regulation of NF-κB regulated genes and inflammatory biomarkers, IHC analyses were carried out on both A549 and PC-3 tumour xenografts biopsies (Supplementary Figures 2 & 3). In both A549 and PC-3 tumour sections, increases in pro-apoptotic and cell cycle regulator protein levels were observed in p21, cleaved caspase-3 and p300, while angiogenic biomarker vascular endothelial growth factor (VEGF) and inflammatory biomarkers histone deacetylase 2 (HDAC2), cyclooxygenase-2 (COX-2) and 5-lipoxygenase (5-LOX) were significantly reduced compared to placebo levels (Figure 6). An over expression of HDAC2, COX-2 and 5-LOX have been implicated in the growth and progression of various cancer types, and are also found to be regulated by the NF-κB pathway [18-20]. Cyclin-dependent kinase inhibitor, p21 plays an important role in preventing tumour development, whereby inducing p21 has been shown to cause cell cycle arrest [21]. The p50/p65 complex is the most common and dominant NF-κB heterodimer form in most cancer types [22]. From IHC data, p65 (RelA) NF-κB subunit mean protein levels were found to be highly expressed in tumour tissue from placebo sections (152.85 to 154.39) and in CDDP treated sections (150.82 to 153.80) compared to low expression in ACA treated sections (119.95±14.71), rhAFP treated sections (139.60±6.42), 1:1 ratio (116.04 to 119.92), 1:3 ratio (118.76 to 119.98) and 1:5 ratio treated sections (123.03 ± 8.14). Both PC-3 and A549 section indicated similar reduction patterns in p65 levels as well as other NF-κB regulated genes following rhAFP/ACA treatment, thus supporting the occurrence of NF-κB down-regulation (Figure 6). Overall, similar expression patterns *in vitro* and *in vivo*, as well as between both cancer types demonstrated that the rhAFP/ACA complex modulates its anti-cancer effects consistently. It was also interesting to note that changes in protein levels were most prominent when a 1:1 molar ratio of rhAFP/ACA complex was used.

### Treatment of rhAFP/ACA complex reduces the expression of NF-κB regulated genes and inflammatory biomarkers

**Figure 7:**
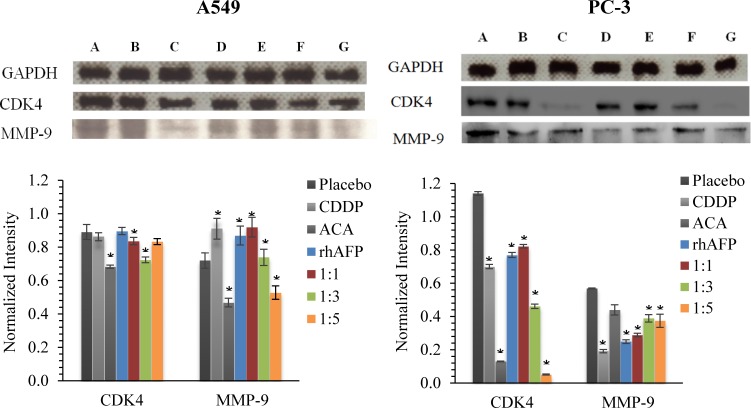
Western blotting analysis of CDK4 and MMP-9 levels Western blotting analysis of CDK4 and MMP-9 levels in A549 and PC-3 tumour biopsies derived from placebo, CDDP treated, ACA stand alone treated, rhAFP stand alone treated, rhAFP/ACA 1:1 ratio treated, rhAFP/ACA 1:3 treated and rhAFP/ACA 1:5 ratio treated groups. GAPDH was used for normalization of band intensities using the ImageJ v1.43 analysis software. All values shown are mean values ± S.D. Statistically significant changes against placebo groups are denoted as (*) with a *p* ≤ 0.05 threshold.

**Figure 8:**
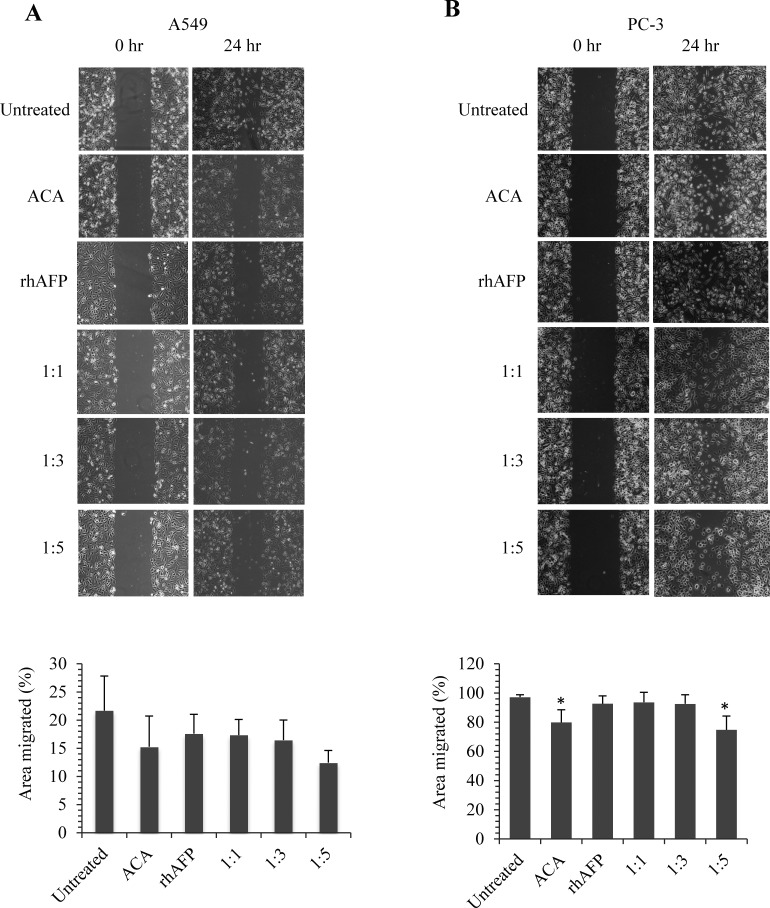
The inhibition effects of ACA stand alone, rhAFP stand alone, and rhAFP/ACA complex on cell migration Representative images at 100× magnification of wound healing assays performed on **A.** A549 cells and **B.** PC-3 cells upon various rhAFP/ACA treatment ratios to assess anti-migration effects. Comparison of percentage area migrated is presented as mean ± S.D. from three replicates using the TScratch software, Version 1.0 (MathWorks Inc., USA). Significant differences in area migrated compared to placebo groups are marked with (*), denoting *p*-values ≤ 0.05.

**Figure 9:**
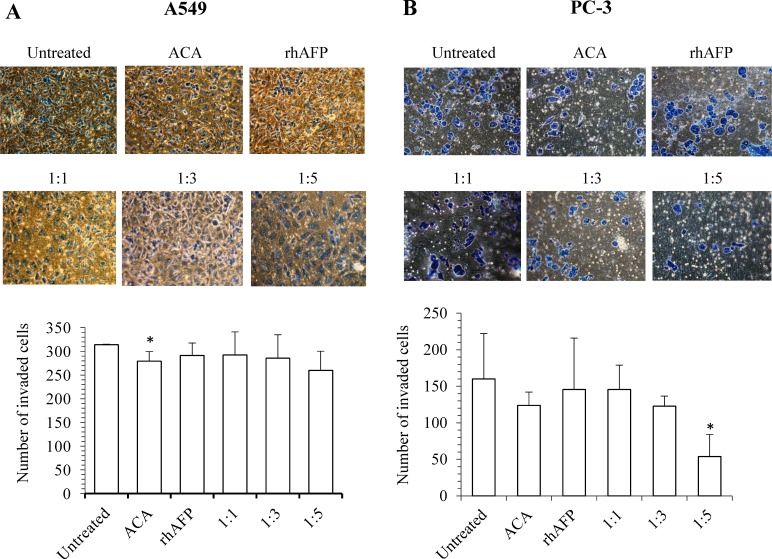
The inhibition effects of ACA stand alone, rhAFP stand alone, and rhAFP/ACA complex on cell invasion Representative cell fields of methylene blue stained invaded cells on the bottom membrane of Matrigel transwell invasion insert for **A.** A549 cells and **B.** PC-3 cells at 200× magnification. Bar graphs representing the average number of invaded cells per field of A549 and PC-3 cells are also shown as mean ± S.D from three independent experiments. Statistically significant differences in comparison to placebo group are denoted by (*) with a *p*-value ≤ 0.05 threshold.

To further assess the effects of NF-κB dysregulation upon rhAFP/ACA treatment, Western blotting was conducted on cyclin-dependent kinase 4 (CDK4) and matrix metalloproteinase-9 (MMP-9). CDK4 is a catalytic subunit of the protein kinase complex of the CDK family that is important for G_1_ phase cell cycle progression. Over expression of CDK4 has been shown in many tumour types including NSCLC, and has been implicated as a key factor in promoting the initiation and development of tumours [23-24]. Our results indicated that protein levels of CDK4 in A549 tumour biopsies were marginally reduced in ACA treated, rhAFP/ACA 1:3 ratio and rhAFP/ACA 1:5 ratio groups compared to other treatment groups. However, in PC-3 tumour biopsies, reduction in CDK4 levels were much more prominent in ACA stand alone treated, rhAFP/ACA ratio 1:3 and ratio 1:5 treated groups (Figure 7). MMPs represent the most prominent family of proteinases associated with tumourigenesis, and have been implicated in cancer invasion and metastasis. In addition to their role in the breakdown of extracellular matrix and cancer cell migration, MMPs regulate signalling pathways that control cell growth, inflammation, or angiogenesis and may even work in a non-proteolytic manner. In tumours, MMP-9 expression has been attributed to infiltrating inflammatory cells, and is regulated by the NF-κB pathway [25]. Western blotting data on MMP-9 revealed that placebo treated groups demonstrated the highest expression levels compared to other treatment groups in PC-3 tumour biopsies (Figure 7), and that ACA in combination with rhAFP was capable of inhibiting cellular migration and invasion (Figures 8 & 9). However, the same findings in PC-3 cells were not observed in A549 cells, where its migration and invasion properties were insignificantly affected by rhAFP/ACA treatment. Both CDK4 and MMP-9 patterns of expression were consistent with IHC analyses, confirming that NF-κB regulated genes were down-regulated upon rhAFP/ACA treatment.

### Efficacy of rhAFP/ACA complex correlates with AFP-R levels

**Figure 10:**
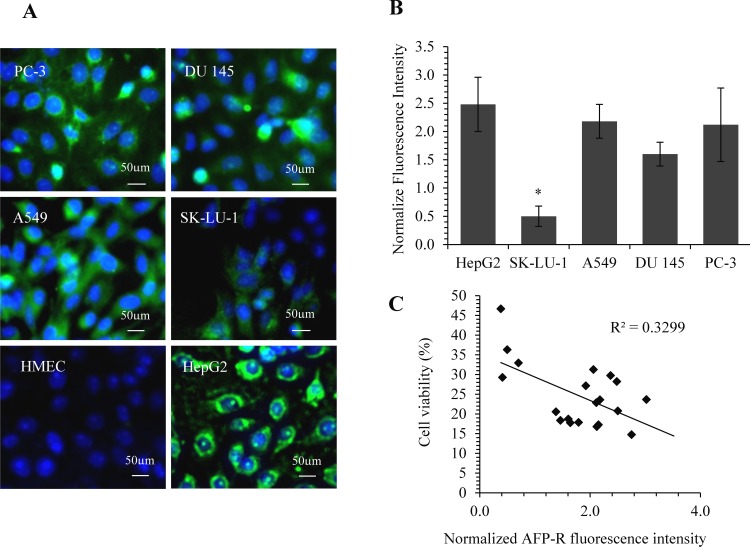
Quantification of AFP-receptor levels using IF staining and its correlation with rhAFP/ACA cytotoxicity data **A.** Representative microphotograph of cells stained with DAPI (blue fluorescence) and anti-AFP-receptor IgG (green fluorescence). All images were captured at 400× magnification with HepG2 as positive control and HMEC cells as negative control. **B.** Comparison of normalized fluorescence intensities value of AFP-R between cell lines. Data presented as mean ± S.D. from three replicates, and were normalized against their respective DAPI intensities. Significant differences in AFP-R levels between each cell line are marked with (*), denoting *p*-values ≤ 0.05. **C.** Dot plot indicating a weak negative correlation (*R*^2^ and *R* value) between AFP-R levels and rhAFP/ACA treated cell viability data among various cancer cell lines tested (*n* = 4).

In order to determine whether the efficacy of rhAFP/ACA regimes correlate with levels of AFP-receptors on tumour cells, IF-IC analyses were carried out on two representative NSCLC and prostate cancer cell lines respectively. Our data suggested a weak negative correlation between cell viability levels and AFP-R levels with a correlation coefficient value (*R*) of −0.57 and *R^2^* value of 0.33 (Figure 10). Additionally, it was found that AFP-Rs were present at varying levels among tumour cell types with HepG2 positive control cells indicating the highest level of receptors, and were almost entirely absent in non-tumour cells as indicated by anti-AFP-R IgG binding. This supported the use of rhAFPs to augment ACA's specificity towards tumour cells, and to a certain extent, also explain the reduced side effects observed *in vivo* in comparison to ACA and CDDP stand alone treatments.

**Table 2:** CI values calculated from MTT cell viability assays after various *in vitro* combination treatments with ACA and rhAFP for 24 h and 48 h

Cell lines	Treatment regimes	Treatment period (h)	CI values	Drug relationship
A549	Constant rhAFP : Variable ACA^††^	24	0.54 ^†^	Synergistic ^†^
		48	0.60 ^†^	Synergistic ^†^
	Constant IC25 ACA : Variable rhAFP	24	1.29	Antagonistic
		48	1.60	Antagonistic
PC-3	Constant rhAFP : Variable ACA ^††^	24	1.42	Antagonistic
		48	1.08	Antagonistic
	Constant IC25 ACA : Variable rhAFP	24	0.88	Synergistic
		48	0.76 ^†^	Synergistic ^†^

† Significantly synergistic values

†† rhAFP concentrations constant at 5.0 μM

Data are presented as mean ± SD of independent triplicate experiments. A CDI value of less than a 0.8 threshold indicates that the drugs are significantly synergistic.

## DISCUSSION

One of the central challenges of modern-day chemotherapy is the design of drugs displaying high selectivity towards tumour cells. In an effort to improve the efficacy of cytotoxic agents without increasing the burden of side effects, researchers have developed strategies to prevent easy diffusion by binding these toxic drugs to macromolecules, such as antibodies, serum proteins, lectins, peptides, growth factors and synthetic polymers. One important aspect that should be taken into consideration when using these carriers is that the process of crossing the cell membrane should be made as specific as possible [26]. Since rhAFP has the ability to bind various water insoluble small molecules within its hydrophobic pockets, it is a suitable drug carrier candidate of ACA. This allowed the solubilisation of ACA in an aqueous environment corresponding to human blood, and to function as a targeted delivery vector selectively to tumour cells whilst avoiding normal healthy tissue. Data from adiabatic scanning microcalorimetry and thermodynamic parameters provided evidence that the tertiary structure of the rhAFP molecule is stabilized upon binding to ACA but undergoes significant destabilization induced by its removal, hence confirming its reversible conformational change. It also demonstrates that rhAFP forms a non-covalent complex with ACA leading to the formation of a more stable tertiary structure resistant to heat denaturation. Similarly as previously reported, high concentrations of hydrophobic ligands such as fatty acids and estrogens are able to induce multiple conformational transition forms, but which are reversible, in the tertiary structure of rhAFP [27]. As this study was carried out with rhAFP/ACA molar ratios of up to 1:5, it also suggest that a single rhAFP protein may possess at least five or more binding sites for ACA on its surface allowing simultaneous binding of multiple ligands.

While both rhAFP and ACA stand alone regimes showed tumouricidal effects, their combined use at low concentrations demonstrated a more significant tumour suppressive effect. This is advantageous in terms of addressing dose related toxicity, and is reflected by increased body weight gains in mice models. Furthermore, the ability of rhAFP to accumulate at tumour sites has been recently proven in the past, thus providing highly specific and effective tumour-suppressive effects [28]. In the three molar ratios of rhAFP/ACA complex, pulmonary inflammation was completely absent in the 1:1 ratio, indicating a reduction in side effects due to rhAFP's tumour targeting abilities compared to mice treated with the commercially available CDDP therapeutic regimen. In addition to offering potential oxidative susceptibility protection to ACA via its hydrophobic pockets, rhAFP also allows for a more specific targeting of cancer cells by chaperoning ACA towards malignant cells which are positive for the AFP cell surface receptor. This reduces the effective dose required *in vivo* in comparison to non-specific cytotoxic drugs such as CDDP, therefore minimizing unnecessary damage towards normal tissue. Even though *in vitro* cytotoxicity assays revealed that a 1:5 rhAFP/ACA molar ratio dose induced the highest level of toxicity, our *in vivo* studies indicated that a high 1:5 dose resulted in undesired pulmonary inflammatory side effects. Therefore, on this basis, the recommended molar combination of this complex is the 1:1 ratio which indicated the optimum ratio between free hydrophobic pockets on rhAFP with ACA.

While the innovative use of small molecules conjugated to AFP was not the first of its kind, it is important to note that this is the first time that a recombinant version of human AFP was used. The employment of AFP as a hydrophobic carrier was previously demonstrated with doxorubicin using glutaraldehyde and poly(amidoamine) dendrimers nanoparticles as a cross-linking agents. It was found that the anti-tumour activity of doxorubicin included in the conjugate was significantly higher than that of free doxorubicin, and was attributed to AFP-doxorubicin's specificity for tumour cells and also towards specific features of doxorubicin's entrance and compartmentalization into the cell [29].

The NF-κB family is a key player in controlling both innate and adaptive immunity. Even though NF-κB activation is required for proper immune system function, an inappropriate activation or dysregulation of NF-κB can mediate inflammation and tumourigenesis [30]. High levels of NF-κB pathway activation in tumour cells typically lead to the enhanced resistance towards apoptosis. Therefore, any factors capable of producing inhibitory effects on NF-κB pathway activation are considered as potential sensitizing factors for chemotherapeutic drugs. In our previous study, we have reported that ACA inhibited both canonical and non-canonical NF-κB activation by preventing IKKα/β phosphorylation and IκBα degradation, thereby reducing the expressions of NF-κB gene products such as proinflammatory COX-2 and proliferative cyclin D1 [4]. In present study, we found that rhAFP/ACA can mediate anti-tumour activity by modulating the constitutively active NF-κB subunit, p65, known to regulate the expression of inflammatory proteins [31]. This showed that ACA's mode of action in combination with rhAFP was consistent with our previous *in vitro* data where IKK activation was also suppressed by ACA

Invasion and angiogenesis are critical events for tumour metastasis, and are partly regulated by the NF-κB pathway. While the extent of angiogenesis and invasion *in vivo* were not fully assessed in this study, initial metastasis biomarkers, such as COX-2, 5-LOX, HDAC2, VEGF and MMP-9, did reveal that treatment with the rhAFP/ACA complex successfully down-regulated their protein levels. The rhAFP/ACA treatment up-regulated histone acetylase p300 and cell cycle inhibitor p21 protein expressions while down-regulating histone deacetylase HDAC2 and cyclin dependent kinase CDK4, all of which are important cell cycle regulator proteins [32-34]. Since ACA induces apoptosis through the extrinsic pathway while rhAFP ultimately affects the intrinsic pathway as previously described [4, 17], treatment with this complex would combine these two death pathways as a multimodal therapy to hypothetically enhance the apoptotic effects while reducing the likelihood of therapeutic resistance as seen with single mechanistic drugs such as CDDP.

## CONCLUSIONS

In the present study, the novel and improved pharmaceutical formulation based on tumouricidal plant-derived compound ACA and rhAFP produced in *S. cerevisiae* was described. This advantageous two-component, biochemotherapy and non-covalent complex is recommended for targeting both NSCLC and prostate cancers, and may be extended towards various other types of AFP-R positive human tumours.

## MATERIALS AND METHODS

### Plant material

Rhizomes of *Alpinia conchigera* Griff were collected from Jeli province of Kelantan, east-coast of Peninsular Malaysia. The sample was identified by Prof. Dr. Halijah Ibrahim from the Institute of Biological Science, Division of Ecology and Biodiversity, Faculty of Science, University of Malaya. Extraction of ACA involved solvent extractions, chromatographic methods, HPLC profiling and NMR structure verification according to previous methods [3]. A voucher specimen (KL5049) was deposited in the Herbarium of Chemistry Department, Faculty of Science, University of Malaya.

### Production and isolation of rhAFP

Recombinant human AFP was supplied by Prof. Dr. Elena Dudich and Dr. Eduard Tatulov from Biological System LLC, Institute of Immunological Engineering, Lyubuchany, Moscow, Russia. The production of glycosylated and non-glycosylated rhAFP secreted by *Saccharomyces cerevisiae* was obtained from plasmid transfection of the AFP gene in yeast cells and also those produced by genomic integration of the AFP gene. All corresponding isolation procedures were patented and reported earlier [11-12]. Pilot scale fermentation of rhAFP-secreting yeast strains were carried out in a 5.0 L Biostat B (B. Braun Biotech International, Germany) bioreactor. During fermentation, replenishment with YPG medium (3% yeast extract, 6% peptone, 10% glucose) was continuously performed. In the case of culturing in high density media, the content of rhAFP was quantified using a modified commercial ELISA kit Cat#K225 (Xema-Medica, Moscow, Russia), with yields as high as 100 to 200 mg/L.

### Preparation of non-covalent complexes of rhAFP and ACA

Ligand-free defatted rhAFP was prepared by charcoal/HCl treatment in 0.1 mM acetate buffer at pH 4.0. Just after absorption, AFP preparation was adjusted to pH 7.5 by the addition of concentrated 2M Tris buffer (Bio-Rad, USA) and thereafter dialyzed against phosphate buffered saline (PBS) (MediaTech, USA). Non-covalent complexes of rhAFP with ACA were prepared by 2 h incubation of the protein solution in PBS (10.0 mg/ml) with equimolar amounts of ACA ligands dissolved in DMSO (Merck, Germany) at either (1:1), (1:3) or (1:5) at 25°C. The rhAFP/ACA complexes were used for cell culture or microcalorimetry experiments. The final concentration of polar solvents in cell culture did not exceed 1% (v/v) and was subtracted as a control in cell viability experiments. All unbound ligands were removed by dialysis before microcalorimetry experiments. Calorimetric measurements were performed using a differential adiabatic scanning microcalorimetry (DASM)-4 differentials capillary scanning calorimeter equipped with cells of 0.464 ml working volume. Calorimetric runs of the samples were carried out within a temperature range of 1 to 100ÐC at a heating rate of 1.0 K/min. The specific excess heat capacity function C_p,exe_(T) and specific denaturation heat Q_d_ were calculated as described in [15].

### Cell lines and culture conditions

Human non-small cell lung cancer cell lines (A549 and SK-LU-1), human androgen-independent prostate cancer cell lines (PC-3 and DU 145), human cervical carcinoma cell (Ca Ski), human oral squamous carcinoma cell (HSC-4), human hepatocyte carcinoma cell (HepG2) and normal cell controls human mammary epithelial cell (HMEC) (Lonza Inc., USA) were used. All the cell cancer lines were purchased from American Type Culture Collection (ATCC) except for SK-LU-1 cell lines was purchased from Aseacyte, Malaysia. A549, SK-LU-1, Ca Ski, HSC-4 and HepG2 cells were cultured in Dulbecco's Modified Eagle's Medium (DMEM) whereas PC-3 and DU 145 cells were cultured in Rosewell Park Memorial Institute 1640 (RPMI) medium supplemented with 10.0% (v/v) fetal bovine serum (FBS). While HMEC cells were cultured in Mammary Epithelial Growth Medium (MEGM). All cells were maintained in an incubator at 37ºC in a 5% CO_2_ atmosphere and 95% humidity level.

### Cytotoxic combination effects of rhAFP/ACA compositions *in vitro*

Combinations of ACA and rhAFP at various molar ratios were tested for their killing effects on A549, SK-LU-1, PC-3, DU 145, HepG2, Ca Ski, HSC-4 and human mammary epithelial (HMEC) cells *in vitro* with the MTT assay. Both ACA and rhAFP molar concentrations were optimized to the extent that it would not generate an extensive cytotoxic effect in stand alone treatment (≤ IC_50_). Human tumour and normal cells were seeded in 96-well plates at a density of 1.0×10^4^ cells per well in 100.0 μl serum free medium for 24 h to allow adherence. Various combined molar ratios of rhAFP with ACA ranging from 1:1 to 1:5 were prepared and added into each well. After 24 h of incubation, MTT assays were performed to determine the viability of treated cells. Assessment on the type of combination relationship was done using an isobologram analysis, while the degree of synergy was assessed based on calculated combination index (CI) values, where CI values of >1.0 implies antagonism, 1.0 implies additivity, and <1.0 implies synergistic type relationships between two components. All calculations were based upon the CI equation adapted from previous literature [16].

### *In vivo* efficacy of rhAFP/ACA formulations

Six weeks-old male athymic nude mice (*Nu/Nu*) (Biolasco Taiwan Co. Ltd., Taiwan) were used to assess the effects of rhAFP/ACA formulations *in vivo*. Two sets of studies to evaluate the formulations' therapeutic effects on NSCLC and prostate tumour xenografts were performed simultaneously. Tumour xenografts were induced by injecting 100.0 μl suspensions of A549 or PC-3 cells (5.0×10^7^ cells/ml) in 1×PBS and BD Matrigel Matrix HC subcutaneously (s.c.) at the lateral neck region for the former or the lateral thigh region for the latter using 25 gauge needles. All drugs were prepared in 0.9% (w/v) NaCl solution and administered intraperitoneally (i.p.) when tumour load reached a 100.0 mm^3^ threshold or higher. The following treatment groups (n=6) were assigned: (i) placebo (0.9% NaCl), (ii) CDDP stand alone (10.0 mg/kg), ACA stand alone (1.56 mg/kg), rhAFP stand alone (5.0 mg/kg), rhAFP/ACA 1:1 molar ratio (0.52 mg/kg:5.0 mg/kg), rhAFP/ACA 1:3 molar ratio (1.56 mg/kg:5.0 mg/kg), and combination rhAFP/ACA 1:5 molar ratio (2.6 mg/kg:5.0 mg/kg). Treatments were done biweekly with a 2-3 day interval over an 8-week period inclusive of the tumour induction period. Tumour volumes were measured with a traceable digital calliper (Thermo Scientific, USA) by calculating [(major diameter) × 0.5 × (minor diameter)2] once a week throughout the entire experiment. Net body weight (minus tumour weight) was measured concurrently with tumour volume measurements. Mice were allowed to live an additional 2 weeks post-treatment period to evaluate tumour recurrence rate. Blood samples were collected via tail vein bleeding once a week for quantification of tumour markers. All mice were sacrificed at the end of the study using purified CO_2_, and biopsies of tumours were harvested and fixed in formalin solution. All procedures for animal experimentation were approved by The Institutional Animal Care and Use Committee, University of Malaya [(UM IACUC), ethics reference no: GEN/29/06/2012/NMA(R)].

### Quantification of AFP-receptors using immunofluorescence

Cells were cultured on cover slips placed in 6-well plates overnight followed by removal of spent media and washing with 1× PBS before proceeding with immunofluorescence (IF) staining. Fixation was done using ice-cold methanol for 5 min, and blocked using 10% (v/v) of FBS at 37°C for 20 min. Binding of AFP-receptors were done using mouse anti-AFP-R IgG (Santa Cruz Biotechnology, TX, USA) at 1:50 dilution for 1 hr, while detection of primary antibodies were done using goat anti-mouse IgG conjugated with fluorescein isothiocyanate (FITC) (Santa Cruz Biotechnology, TX, USA) at 1:400 dilution for 1 hr in the dark. Counterstaining of nuclei were done using 4′,6-diamidino-2-phenylindole (DAPI) (Thermo Fisher Scientific, IL, USA) staining for 5 min. All wells were washed three times with 1× PBS for 5 min between each step. Cover slips containing stained cells were mounted on microscope slides using UltraCruz™ Mounting medium (Santa Cruz Biotechnology, TX, USA) and sealed with nail polish. FITC images were captured at 494nm/518nm while DAPI images were captured at 358nm/461nm using the Axio vert.A1 inverted fluorescence microscope (Zeiss, Germany) at 400× magnification. FITC fluorescence intensities were normalized against DAPI intensities as obtained using the Zen 2012 fluorescence intensity quantification software (Zeiss, Germany).

### Immunohistochemistry (IHC) analysis of tumour biopsies

Formalin fixed paraffin embedded (FFPE) tumour sections were subjected to de-paraffinization and rehydration using a graded alcohol series (Cell Signalling, USA). Epitope retrieval was achieved by boiling the tissue sections in sodium citrate buffer (0.01 M, pH 6.0) for 10 min. Endogenous peroxidase activity was blocked using 3% (v/v) hydrogen peroxide. All sections were blocked with Tris-buffered saline with Tween-20 (TBST) and 5% (v/v) normal goat serum (Cell Signalling, USA) for 1 h. IHC was performed using antibodies specific for NF-κB p65 (1:400), COX-2 (1:200), 5-LOX (1:50), VEGF (1:100), p21 (1:50), cleaved caspase-3 (1:800), HDAC2 (1:100) and p300 (1:100), and incubated overnight at 4°C. SignalStain^®^ Boost IHC Detection Reagent (HRP-conjugated mouse or rabbit IgG) (Cell Signalling, USA) was used for signal detection with 3,3′-diaminobenzidine (DAB) solution (Sigma-Aldrich, USA) according to the manufacturer's protocol. Counter-staining was done using hematoxylin (Sigma-Aldrich, MO, USA) and thoroughly washed in dH_2_O. The slides were dehydrated by soaking in a graded alcohol series and cleared by soaking in xylene. Slides were then mounted and sealed using dibutyl phthalate xylene (DPX) mounting medium. Images were captured using an inverted microscope Nikon Eclipse TS 100 (Nikon Instruments, Japan) and quantified using the Nikon NIS-BR Element software (Nikon Instruments, Japan).

### Western blot analysis

Proteins were extracted from tumour biopsies using the Qproteome FFPE Tissue Kit (Qiagen, Germany) according to manufacturer's protocol. Protein concentration was quantified and normalized using the Quick Start Bradford Protein Assay Kit 2 (Bio-Rad, USA) according to manufacturer's protocol. Protein were separated on an SDS-PAGE and transferred to a 0.2 μm nitrocellulose membrane using the TransBlot-SD Semi Dry Transfer Cell (Bio-Rad, USA). Blots were blocked with 5% w/v BSA, 1×TBS, 0.1% Tween-20 at room temperature with gentle shaking for 90 min, and incubated with primary antibodies: GAPDH (1:1000), CDK4 (1:1000), MMP-9 (1:1000) overnight at 4°C. Detection of bound antibodies were done using HRP-conjugated secondary antibodies (Cell Signalling, USA), and SuperSignal West Pico chemiluminescent substrate. Images were captured using the Fusion FX7 imaging system (VilberLourmat, France). Normalization of protein concentration was carried out against GAPDH as a control. Relative intensities of all bands were quantified using ImageJ v1.43 analysis software (NIH, USA).

### Quantification of tumour markers using enzyme-linked immunosorbent assay (ELISA)

Serum levels of carcinoembryonic antigen (CEA) and prostate specific antigen (PSA) were assessed weekly using sandwich ELISA kit (MP Biomedicals, USA) according to manufacturer's protocol. Briefly, samples were immobilized with goat antibody (zero buffer) before addition of monoclonal HRP-conjugated anti-PSA IgG or anti-CEA IgG for 2 h at room temperature. Detection was done using 3,3′,5,5′-tetramethylbenzidine (TMB) solution in the dark at room temperature for 30 min. Colour development was stopped by adding HCl stop solution, and absorbance values were measured spectrophotometrically at 450 nm wavelength. A linear standard concentration range (0 ng/ml to 120 ng/ml) was established for both PSA and CEA which was used to correlate absorbance values to concentration values.

### Migration assay

Cell migration was determined using the wound healing assay. Equal number A549 or PC-3 cells (4.0 × 10^5^/ml) were seeded in 6-well plates and incubated at 37°C in 5% CO_2_ for 24 h in growth media with 10% FBS (Kansas, USA) media to allow cells to attach onto the plate to form a monolayer. The growth media was changed to serum-free medium containing of Mitomycin-C (Calbiochem, USA) at 1.0 μg/ml and further incubated in 37°C for 2 h to inhibit cell proliferation, before wounds of similar size were introduced into the monolayer by a sterile pipette tip. Cell debris generated from the scratch were washed with 1x PBS twice, and treated with ACA stand alone, rhAFP stand alone, or combination of rhAFP and ACA in serum-free medium for 24 h at 37°C. The images and speed of wound closure was documented at 0 h and 24 h post-wounding using the Nikon Eclipse TS100 inverted fluorescence microscope (Nikon Instruments, Japan) and analyzed using TScratch software, Version 1.0 (MathWorks Inc., USA).

### Transwell invasion assay

Cell invasion capacity of selected sub-cell lines were examined using transwell invasion assay by measuring the number of cells transmigrating through a layer of extracellular matrix, Matrigel. Equal number A549 or PC-3 cells (1.0 × 10^5^/ml) were seeded in 6-well plates and were treated with ACA stand alone, rhAFP stand alone, or combination of rhAFP and ACA for 24 h at 37°C. 24-well transparent PET membrane 8.0 μm pore size insets were coated with 70.0 ul of 1.5 mg/ml Matrigel (BD Biosciences, USA). Cells were starved with serum free media and harvested after 20 h. Cells were resuspended with 500.0 μm of serum free media and were added to the upper insert, whilst media with 20.0% (v/v) FBS was added at the lower insert as a chemo-attractant. The cells were incubated for 24h at 37^o^ C. Cells in the upper insert were removed with cotton swabs, and invading cells on the underside of the membrane were fixed in 100.0% ethanol for 2 min, followed by staining with 1.0% (w/v) methylene blue (Sigma, USA) for 20 min. Number of invaded cells in eight random fields of each transwell invasion membrane insert area were counted under the Nikon Eclipse TS100 inverted microscope (Nikon, Japan) at 200× magnification.

### Statistical analysis

Data from all experiments were presented as mean ± SD of at least three to six replicates. Differences between samples were considered statistically significant with *p*-values ≤ 0.05 as calculated using paired Student's T-test.

## SUPPLEMENTARY MATERIAL FIGURES


