# Supplementary material for: Recombinant human alpha fetoprotein synergistically potentiates the anti-cancer effects of 1′-S-1′-acetoxychavicol acetate when used as a complex against human tumours harbouring AFP-receptors
Source: Oncotarget. 2015 Apr 29;6(18):16151–67. doi: 10.18632/oncotarget.3951 (PMC4599262; doi:10.18632/oncotarget.3951)
Supplement: Supplementary file 1 [file oncotarget-06-16151-s001.pdf]

Recombinant human alpha fetoprotein synergistically potentiates the anti-cancer effects of 1′-S-1′-acetoxychavicol acetate when used as a complex against human tumours harbouring AFP-receptors

Supplementary Material

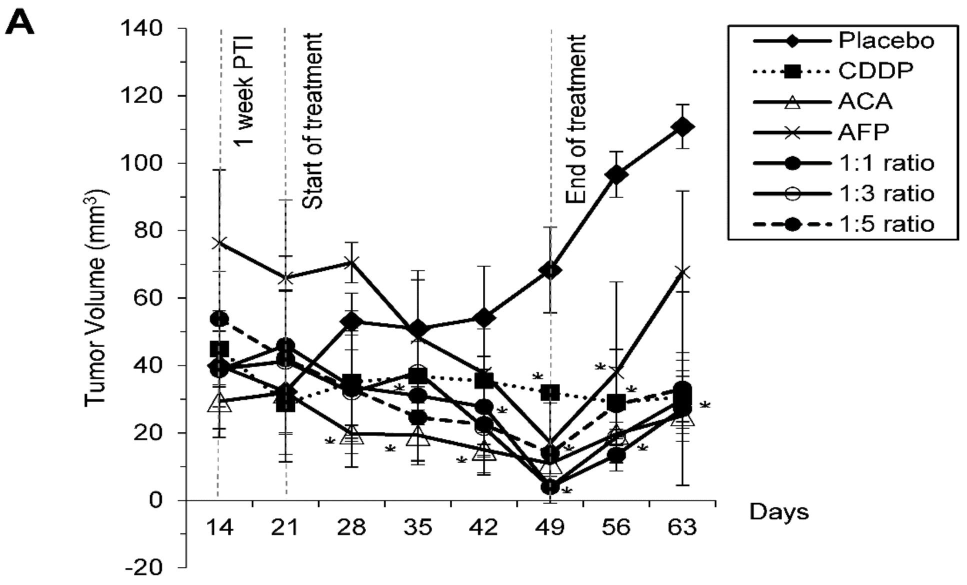

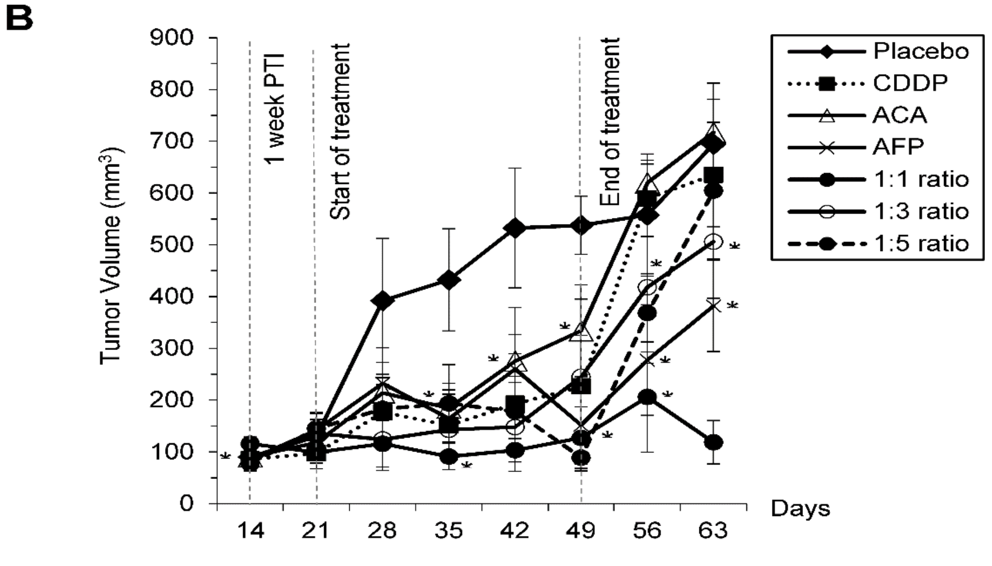

Supplementary Figure 1: Tumour reduction effects of various rhAFP/ACA treatment regimes on Nu/Nu mice. (A) NSCLC (A549) tumour growth curve and (B) Prostate (PC-3) tumour growth curve between various groups over a period of 9 weeks. Mice were allowed to live an additional 2 weeks post-treatment period to evaluate tumour recurrence rate. Mice were

treated with rhAFP/ACA as stand alone and at various concentration ratios 1 week post-tumour implantation (PTI) after tumour volumes of ≥ 100mm3 were achieved. All data shown are mean values ± S.D. Statistically significant changes against placebo groups are denoted as (*) with a $p \leq 0.05$ threshold.

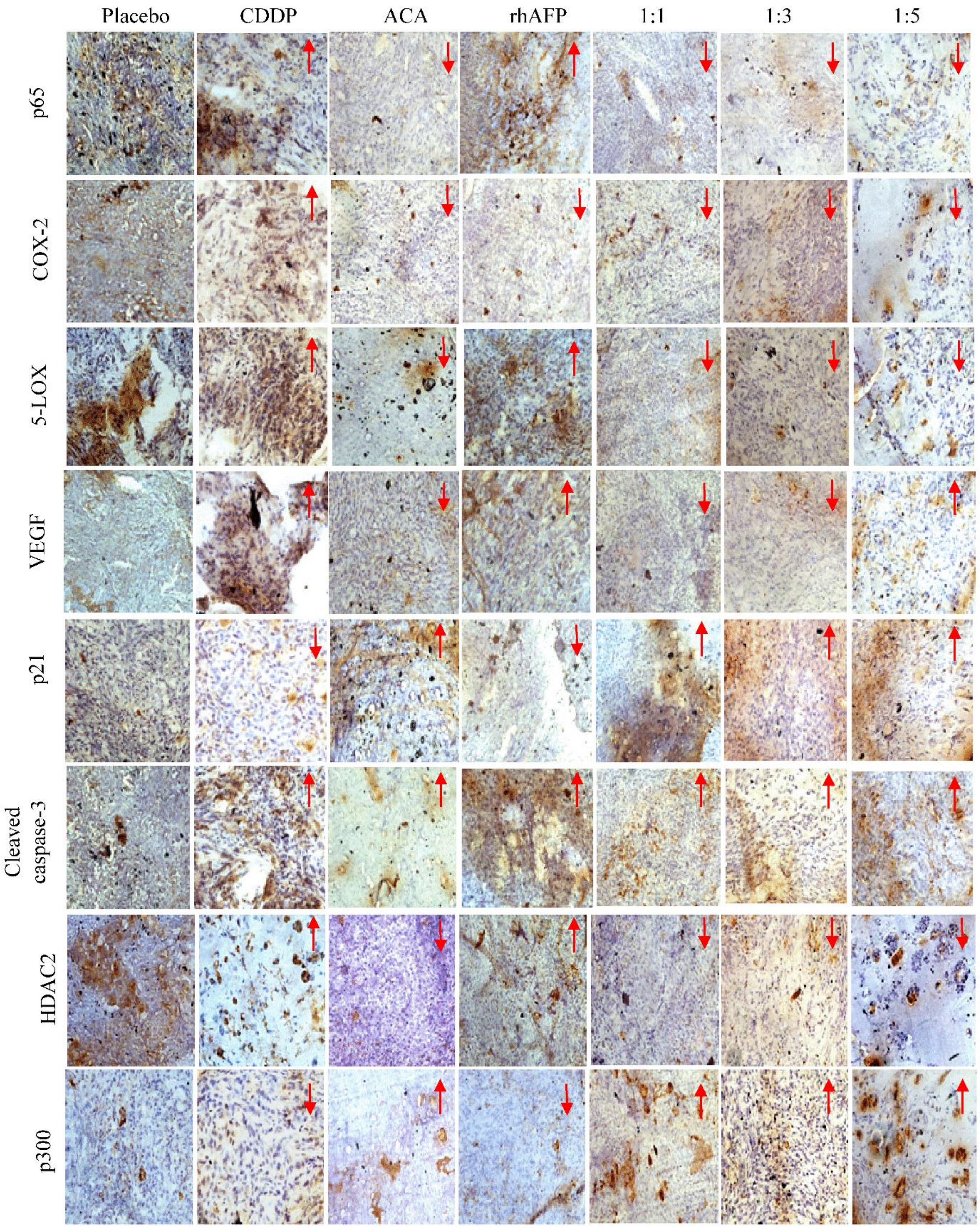

Supplementary Figure 2: IHC analyses on NF-κB regulated proteins and inflammatory biomarkers on A549 xenograft sections treated with various rhAFP/ACA combination regimes. Blue colour indicates nuclei stained with hematoxylin and brown color indicates DAB antibody staining. Arrows indicate up- or down-regulation of proteins in comparison to placebo control. All images were shown as a representative of three independent replicate at 400× magnification

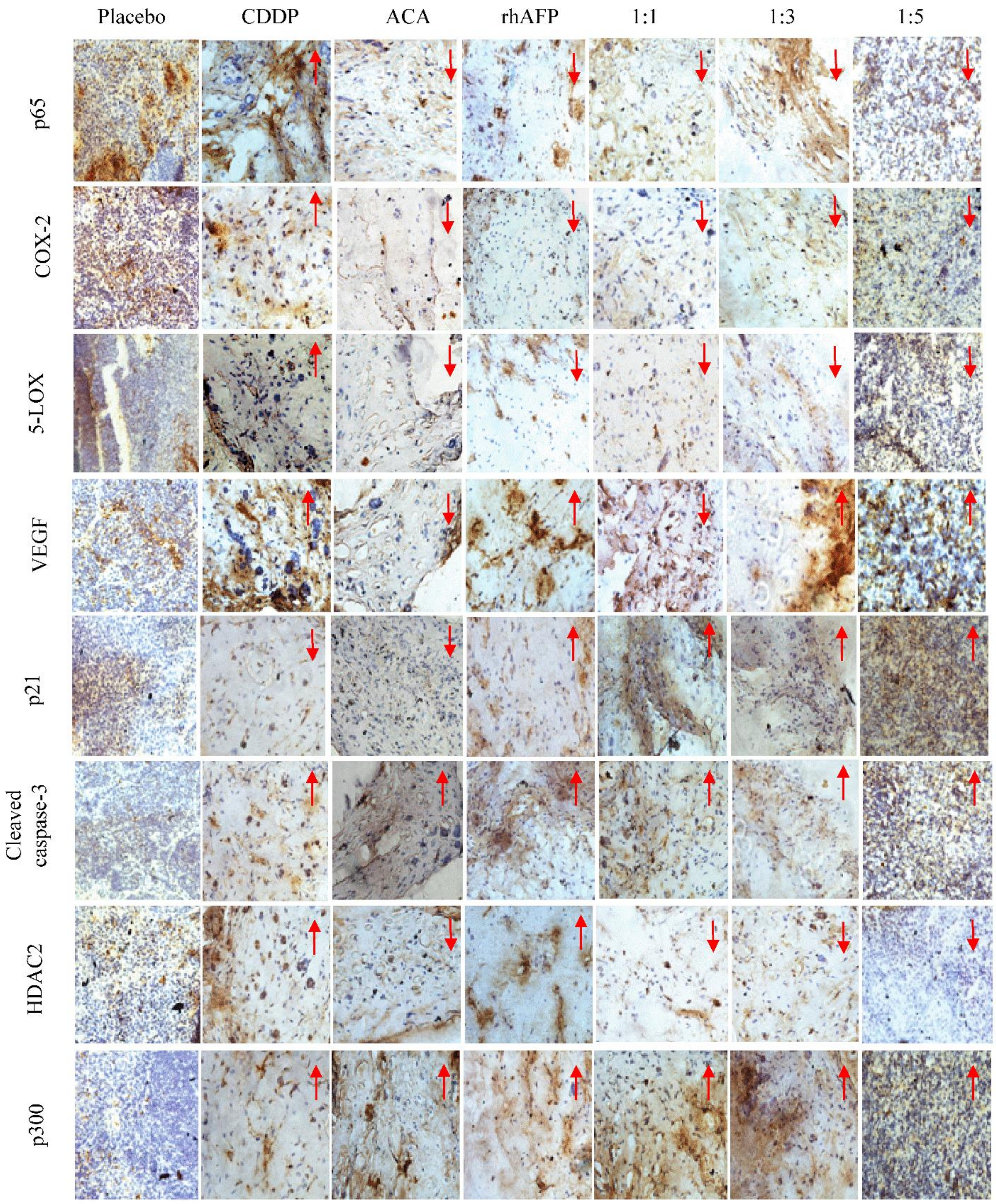

Supplementary Figure 3: **IHC analyses on NF-κB regulated proteins and inflammatory biomarkers on PC-3 xenograft sections treated with various rhAFP/ACA combination regimes.** Blue colour indicates nuclei stained with hematoxylin and brown color indicates DAB antibody staining. Arrows indicate up- or down-regulation of proteins in comparison to placebo control. All images were shown as a representative of three independent replicate at 400× magnification.
